# Defence Chemistry Modulation by Light and Temperature Shifts and the Resulting Effects on Associated Epibacteria of *Fucus vesiculosus*

**DOI:** 10.1371/journal.pone.0105333

**Published:** 2014-10-31

**Authors:** Mahasweta Saha, Martin Rempt, Stephanie B. Stratil, Martin Wahl, Georg Pohnert, Florian Weinberger

**Affiliations:** 1 Department of Benthic Ecology, Helmholtz-Zentrum für Ozeanforschung (GEOMAR), Kiel, Germany; 2 Institute for Inorganic and Analytical Chemistry Bioorganic Analytics, Friedrich-Schiller-Universität Jena, Jena, Germany; CSIR- National institute of oceanography, India

## Abstract

The goals of this study were (1) to investigate whether *Fucus vesiculosus* regulates the production of its antifouling defence chemicals against epibacteria in response to light limitation and temperature shifts and (2) to investigate if different surface concentrations of defence compounds shape epibacterial communities. *F. vesiculosus* was incubated in indoor mesocosms at five different temperature conditions (5 to 25°C) and in outdoor mesocosms under six differently reduced sunlight conditions (0 to 100%), respectively. Algal surface concentrations of previously identified antifouling compounds - dimethylsulphopropionate (DMSP), fucoxanthin and proline – were determined and the bacterial community composition was characterized by in-depth sequencing of the 16S-rRNA gene. Altogether, the effect of different treatment levels upon defence compound concentrations was limited. Under all conditions DMSP alone appeared to be sufficiently concentrated to warrant for at least a partial inhibitory action against epibiotic bacteria of *F. vesiculosus*. In contrast, proline and fucoxanthin rarely reached the necessary concentration ranges for self-contained inhibition. Nonetheless, in both experiments along with the direct influence of temperature and light, all three compounds apparently affected the overall bacterial community composition associated with *F. vesiculosus* since tendencies for insensitivity towards all three compounds were observed among bacterial taxa that typically dominate those communities. Given that the concentrations of at least one of the compounds (in most cases DMSP) were always high enough to inhibit bacterial settlement, we conclude that the capacity of *F. vesiculosus* for such defence will hardly be compromised by shading or warming to temperatures up to 25°C.

## Introduction

Marine intertidal environments are characterised by large variation and abrupt shifts in environmental factors. Extreme conditions like high temperatures, intense sunlight or desiccation occur relatively often, triggering multiple physiological responses in intertidal organisms such as the rockweed *Fucus vesiculosus* [1], [2] and may also cause variation in their capacity for chemical defences [3], [4]. A large body of literature indicates that *Fucus* and other macroalgae are generally associated with more or less dense and highly diverse communities of epibiotic microorganisms [5], [6]. Epiphytic Bacteria can have multiple effects upon their host that can be either beneficial or detrimental [7], [8]. Detrimental effects include – but are not limited to – pathogenicity and attraction of macrofoulers. In order to limit these negative effects algal hosts often develop chemical defence strategies to control the colonisation by unwanted microbial foulers [9], [10].

*Fucus vesiculosus* is known to be capable of chemical defence against microfoulers. Artificial substrates containing surface extracts of this alga maintain microbial communities that are relatively similar to those associated with *F. vesiculosus* [11]. Furthermore, the alga harbours at its surface fucoxanthin, dimethylsulfopropionate (DMSP) and proline, which all reduce bacterial settlement [10], [12]. This activity is strain-specific and while many strains are repelled by those compounds, others can be attracted [12]. Thus, the mentioned repellents not only influence bacterial density but also have the potential to influence epibacterial community composition on *Fucus* as certain strains are attracted and others deterred.

The production of defence chemicals is usually expected to cause energetic costs [13]. For example, the production of the bacterial colonisation inhibitor 1,1,3,3-tetrabromo-2-heptanone by the red alga *Bonnemaisonia hamifera* has been found to be negatively correlated with its growth [14]. Similarly, the production of phlorotannins - confirmed deterrents of certain herbivores [15] and suspected deterrents of fouling organisms [16], [17] – has been reported to be negatively correlated with growth of the rockweed *Fucus vesiculosus* [18]. However, defence compounds like phlorotannins have multiple roles in marine plants; including UV protection.Their production may therefore be driven by other needs than defence [19]. This could be the case for all three confirmed anti-bacterial compounds from *Fucus*, as fucoxanthin is also the main accessory pigment of photosynthesis, while proline and DMSP have osmoregulatory functions [20], [21]. DMSP also acts as an antioxidant [22] and proline is also an essential constituent of most proteins.

Both light limitation stress and temperature shift stress have been shown to limit the capacity of *F. vesiculosus* for induced anti-herbivore defence [3]. The purpose of the present study was to investigate whether there is a similar effect of environmental shifts on the presence of bacterial settlement inhibitors on the algal surface. To answer this question, surface concentrations of fucoxanthin, DMSP and proline were quantified after different temperature and light treatments. To evaluate the potential effect of changing concentrations upon microbial settlers two different approaches were chosen: (1) Detected concentrations were compared to the known necessary doses for half maximal settlement inhibition of potential bacterial colonisers of *Fucus* [10], [12]; and (2) detected concentrations were correlated to the relative abundance of major bacterial groups, that were identified in both experiments by in-depth sequencing of the 16S-rRNA gene. The concentration of mannitol, the main primary energy storage compound of *Fucus*, was used to quantify the degree of light limitation stress.

## Materials and Methods

### General experimental setup

*Fucus vesiculosus* L. was cultivated in two successive monofactorial experiments under independently controlled temperature and light conditions. The effect of different water temperatures at identical light conditions was compared in a “temperature experiment”, while the effect of different light intensities at identical water temperatures was investigated in a “light experiment”. Temperature and light experiments differed in several aspects and hence the results are interpreted within the experiments and there is limited scope to compare them between the experiments.

### Algal material

Whole individuals of *Fucus vesiculosus* were collected from the littoral zone of the Kiel Fjord, Western Baltic (54°27′N/10°11′E for the temperature experiment and 54°23′N/10°12′E for the light experiment) from a depth between 0.5–1 m in October 2009 and March 2011, respectively. The algae were individually sealed in zip-lock bags filled with ambient seawater, transported to the laboratory in a cooler box and subsequently maintained in individual aquaria. No special permission was required to sample *Fucus vesiculosus* from the Kiel fjord.

### Temperature experiment

Twenty-five *Fucus vesiculosus* individuals were maintained separately in 25 indoor aquaria (25 L) in a temperature constant room (15°C) for 28 d. Water was replaced once a week by seawater composed of one third filtered fjord water (16 psu) mixed with two thirds of artificial seawater (Instant Ocean, Blacksburg, VA, 16 psu). This was done to limit diatom growth by nutrient dilution. Five different water temperature levels were tested: 5, 10, 15, 20 and 25°C (±0.5) with n = 5 per treatment level. Five replicate aquaria of each individual temperature treatment were maintained in a large water bath heated or cooled to the desired temperature. Higher temperatures were obtained through aquaria heaters (Schego 600, Schemel & Goetz GmbH and Co KG), while lower temperatures were obtained through coolers (Aqua Medic Titanium 1500, Aqua Medic GmbH, Bissendorf). Water was circulated in the water baths and aquaria with pumps (Aqua EL Circulator 350; Aqua EL GmbH, Germany), in order to ensure homogenous temperatures (see [6] for more details). All aquaria were maintained at a light intensity of 100 µmol m^−2^ s^−1^ (SD ±5), with 8∶16 light∶ dark cycle. This daily dose is well above low-irradiation and below high-irradiation stress levels [3].

### Light experiment

The setup consisted of an outdoor seawater system of 30 aquaria (20 L each). Natural and unfiltered seawater of, on average, 5°C (SD ±0.09) from the Kiel fjord was continuously circulated through these aquaria at a rate of 40 L h^−1^. Side and bottom walls of the aquaria were covered with black plastic bags, in order to exclude diffused light. Six different treatment levels were tested: full sunlight (28 d – mean of 247 µmol m^−2^ s^−1^; SD ±4.24), 44% sunlight (achieved through shading with 1 layer of mosquito gauze (Max Bahr GmbH, Kiel), 23% sunlight (2 layers), 13% sunlight (3 layers), 5% sunlight (4 layers) of natural sunlight and complete darkness (by covering with a black plastic bag). Five replicate aquaria were maintained at each of these light conditions with random spatial distribution. The water temperature and light intensity were logged at 30 min intervals (HOBO, Onset Computer Corporation, USA) in two out of five replicates of each treatment level. The algae were placed individually into these aquaria and acclimatized to the new environmental conditions for 4 days before the start of the experiment. The duration of the treatment was 18 d.

### Quantification of defence chemicals

At the end of each experiment, algal branches of approximately 4–5 cm length (measuring from the tip) were surface extracted by dipping them for 10 s into a stirred mixture of 1∶1 MeOH∶hexane. This method is non-destructive to the epidermal cells of *Fucus vesiculosus* (see [10], online supplementary material). The extracts were vacuum-dried in a rota-evaporator and fractionated into non-polar (hexane) and polar (MeOH) extracts as described in [10] and stored at −20°C until further use. Fucoxanthin was quantified using a Macherey- Nagel (Düren, Germany) Nucleodur analytical normal phase Si column (4.6 mm×25 cm) on a Varian (Palo Alto, Cal.) 940-LC (gradient: 100% n-heptane, 10 min; linear gradient to 100% Ethylacetate for a further 20 min; flow rate 1 ml min^−1^) with integrated photodiode array detector (PDA) at a wave length of 450 nm. A calibration curve of peak areas with eight concentrations of standard fucoxanthin (Cayman Chemicals, Hamburg, Germany) was used. Quantification of DMSP was done by LC-MS according to [23]. Proline quantification was done using the same LC-MS parameters and external calibration with three concentrations.

### Bacterial community composition vs. surface concentration of defence chemicals

In order to test whether concentrations of fucoxanthin, DMSP or proline in surface extracts of *F. vesiculosus* affect the presence or absence of specific groups of bacteria on the algal surface, the bacterial community composition was analyzed by in depth-sequencing of 16SrRNA. Then, the relative abundances of bacterial taxa were correlated to the surface concentration of the three defence chemicals.

DNA samples for the temperature and light treatment were generated after 14 and 10 d of treatment, respectively. Surface extracts were generated from the same *Fucus* individual at the end of the treatment phase in both experiments. Different algal branches of the same individual were used for sampling in order to avoid false compound concentration(s) that may have resulted from the swabbing of the thallus surface for the purpose of bacterial DNA sampling.

The protocols for sampling of the epiphytic bacterial community, for DNA extraction of bacteria, and for 454 pyrosequencing of this DNA for community analysis in the temperature experiment have already been published [6]. Also, the bacterial community in the light experiment was sampled and analysed according to [6] and references therein. Only the number of cycles in the PCR differed (30 cycles in the light treatment instead of 25 in the temperature treatment). Briefly, two young fronds per *Fucus* individual were swabbed with a sterile cotton swab in order to harvest the natural bacterial community. DNA was extracted using the QIAamp DNA Mini Kit (Qiagen GmbH, Hilden, Germany) and was stored at −20°C until 454-pyrosequencing. Here, fragments of ∼450 base pairs (bp) of the V1–V2 hypervariable region of the 16S rRNA gene were amplified. Amplicon libraries were sequenced with a 454 GS-FLX pyrosequencer using the Titanium Sequencing Kit (Roche, Penzberg, Germany) at the Institute of Clinical Molecular Biology (ICMB), Kiel, Germany. Sequences were denoised, grouped at 97% sequence similarity to form OTUs (Operational Taxonomic Units), and classified in the Greengenes 16S rRNA reference database (DeSantis et al. 2006) following the steps in Stratil et al. (2013b). A random subsample of 1024 (light treatment) and 1352 (temperature treatment) high-quality sequences per sample was drawn for further analysis. The detection frequency of OTUs within samples varied and was regarded as an indicator of their relative abundances. Sequences were submitted to the sequence read archive of NCBI under accession numbers SRX204294-SRX204322 (light) and SRX195663-SRX195687 (temperature).

In the temperature experiment the bacterial community composition clearly differed among treatments [6]: approximately 20% of the overall variability among individual samples could be explained by temperature and numerous OTUs occurred only at certain temperature conditions. Compositional variability was also observed among treatments in the light experiment (data not shown). Any indirect treatment effects upon bacteria through modulation of the host's defence behaviour were in both experiments necessarily confounded with direct temperature or light effects upon bacterial growth rates and recruitment/competition between OTUs. Correlations between bacterial relative abundances and concentrations of defence compounds were for this reason only assessed within each treatment level. Spearman correlation coefficients were calculated for each group of five algal replicates of a given treatment level between the relative abundance of each detected OTU and the surface concentrations of fucoxanthin, DMSP and proline. In this way, OTUs that either consistently exhibited negative or consistently exhibited positive correlations between relative abundance and presence of one of the investigated defence compounds could be identified and grouped. For example, in the light experiment 31.1% of all OTUs consistently exhibited a negative correlation between their abundance and presence of proline (hereafter called “Proline-negative”), while 29.9% always correlated positively (“Proline-positive”). The remaining 38.9% of all OTUs exhibited positive correlations in some treatment levels and negative correlations in other levels and were therefore considered as “Proline-neutral”. Similar patterns were also observed in the light experiment for DMSP (“DMSP-positive”, “-negative” and “-neutral”) and Fucoxanthin (“Fucoxanthin-positive”, “-negative” and “neutral”) and in the temperature treatment for all three compounds.

While negative correlations hint at a potentially deterring effect, positive correlations might indicate attraction. Inhibitors of bacterial settlement often only target a limited spectrum of phylogenetic groups of bacteria [9]. We therefore hypothesized that certain phylogenetic clades may be over- or underrepresented within the groups of deterrent-positive and deterrent-negative OTUs, as related to their prevalence within the total microbial community. To test this hypothesis the odds of presence of each clade within deterrent-positive and deterrent-negative subgroups were calculated following [24] as
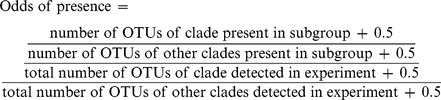



Odds of presence >1 indicate a higher abundance of a given clade within a subgroup than within the total bacterial community, while odds of presence <1 indicate the inverse. Geometric means with 95% confidence intervals were calculated from the odds of presence obtained for each treatment level in the temperature treatment and the light treatment as described in [24]. 95% confidence intervals excluding 1 hint at odds of presence that are significantly larger or smaller than 1 and thereby different between subgroup and total bacterial community. In addition *Χ* ^2^ statistics were used according to [24] in order to test whether the detected abundance of phylogenetic clades within subgroups diverged from their expected abundance ( = their abundance within the total community).

### Quantitative analysis of biofilm

To investigate whether there is an effect of different temperature and light treatments upon the abundance of diatoms and bacteria on the *Fucus* surface, microfoulers were quantified on the 28^th^ day for the temperature experiment and the 10^th^ and 18^th^ day for the light experiment. For the temperature experiment, bacterial cell abundance was determined through direct cell counts at the algal surface at a magnification of 630× after DNA staining with DAPI, using epifluorescence microscopy (details in [6]). For the light experiment, biofilms from 1 cm^2^ of algal surface (1 cm from the tips) were harvested by swabbing with a sterile cotton tip, followed by vortexing the cotton tip for 30 sec in an Eppendorf vial containing 1 ml of sterile-filtered seawater (SSW). The relative abundance of diatoms (and any other possible photoautotrophs) in 100 µL of subsample was determined by measuring the fluorescence of chlorophyll a at 477–491 nm (excitation) and 677 nm (emission), using a plate reader (Hidex Chameleon IV, Turku, Finland) and 96-well microtiter well plates (Greiner). Subsequently, the relative density of all microfoulers (including bacteria and diatoms) was determined by staining all the particles in the same 100 µL subsample with the fluorescent DNA–binding dye Syto 9, 0.005 mM (Invitrogen GmbH). Following an incubation time of 10 min in darkness, fluorescence was subsequently measured (excitation 477–491 nm, emission 540 nm), using the same plate reader. Replication was 10 fold per treatment (n = 9 for bacterial abundance quantification). The first measurements provided data on treatment effects on the relative microalgal density at the algal surface, while the second measurement provided similar information on all epibiotic cells.

### Analysis of mannitol

The mannitol concentration was quantified in order to determine whether light reduction resulted in carbon limitation [3]. After the light experiment, six individuals, each kept at different light treatment level, were freeze-dried, ground and stored at −20°C. Mannitol was extracted and analysed as described in [25], but periodate oxidation was stopped after 10 s.

### Statistical analysis

One-way ANOVA was used to analyse the effects of temperature and light on the DMSP, proline and fucoxanthin concentration separately. One-way ANOVA was further used to analyse quantitative differences in microfouling during the light treatment. Shapiro–Wilk's test was used to test for normal distribution, while Levene's test was used to test for homogeneity of variances. Datasets not fulfilling the criterion of homoscedasticity were Box-Cox transformed using the software Minitab 12.2 (Minitab Inc., State College, PA, USA). Post hoc comparisons of DMSP, proline and fucoxanthin concentration variation among different temperature or light treatments were made using Tukey's honest significant difference (HSD) test (p<0.05). Correlation analysis was used to analyse the relationship between diatom abundance and fucoxanthin concentration. The computer program Statistica (StatSoft, Tulsa, OK, U.S.A.) was used to conduct all statistical tests.

## Results

### Temperature experiment

#### General algal response

After two weeks of incubation, the highest tested water temperature of 25°C visibly exerted stress upon *F. vesiculosus*, as the apical tips in one out of five individuals started to decay. Such decay was detected in four out of five individuals after three weeks and the symptom progressed further, so that mainly old parts were alive in all five individuals at 25°C after four weeks of incubation (data not shown). No decay was observed at 5, 10 and 20°C, while one individual at 15°C also showed the symptom from the 2^nd^ week on, although to a lesser degree.

#### Quantification of defence chemicals

**Figure 1:**
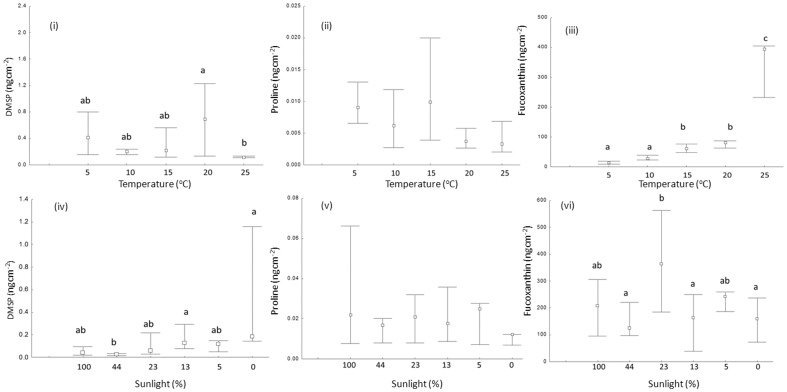
Variation of surface DMSP, proline and fucoxanthin concentration, respectively in *F. vesiculosus* treated under different temperature and light conditions for 28 d and 18 d, respectively. Different letters indicate different temperature and light treatment responses in compound concentration (Tukey's test P<0.05). Median (central symbol), n = 5 (n = 4 for fucoxanthin measurement at 10°C), interquartile range.

In the temperature experiment, healthy apical branches exhibited, on average, surface concentrations of DMSP and proline ranging from 0.16 to 0.96 ng cm^−2^ and from 0.004 to 0.01 ng cm^−2^, respectively. Fucoxanthin concentrations ranged from 18 to 400 ng cm^−2^. However, there was often considerable variability of concentrations within treatment levels (Figs. 1(i) to (iii)). Nonetheless, the surface DMSP concentration also varied moderately between temperature levels (1-way ANOVA, F = 3, p = 0.03) and was higher at 20°C than at 25°C (Tukey's HSD test, p<0.05, Fig. 1(i)). The surface proline concentration did not vary significantly with temperature (1-way ANOVA, p = 0.05, Fig. 1 (ii)), however it tended to decrease with warming. In contrast, the fucoxanthin surface concentration increased significantly with temperature (1-way ANOVA, F = 42, p<0.001, Fig. 1 (iii)), with a five-fold increase between 20°C and 25°C (Table 1, Fig. 1 (iii)).

**Table 1:** Variation in mean surface concentrations of DMSP, proline and fucoxanthin on apical tips of *F. vesiculosus* at different temperature and light conditions.

	*Previously detected concentrations * [10], [12]	*Previously determined EC50*	*Mean concentrations detected [ng cm^−2^ of apical tips] in temperature treatment*					*Mean concentrations detected [ng cm^−2^ of apical tips] in light treatment*					
			*5°C*	*10°C*	*15°C*	*20°C*	*25°C*	*0%*	*5%*	*13%*	*23%*	*44%*	*100%*
**DMSP**	0.12 to 1.08 ng cm^−2^	<0.1 to 0.38 ng cm^−2^	0.46(0.30)	0.17(0.07)	0.59(0.75)	0.96(0.76)	0.16(0.13)	0.45(0.43)	0.1(0.04)	0.17(0.10)	0.1(0.09)	0.03(0.16)	0.3 (0.57)
**Proline**	0.09 to 0.59 ng cm^−2^	0.01 to 0.13 ng cm^−2^	0.009(0.002)	0.006(0.003)	0.01(0.006)	0.004(0.001)	0.004(0.001)	0.01(0.006)	0.025(0.01)	0.02(0.04)	0.03(0.12)	0.02(0.17)	0.03(0.39)
**Fucoxanthin**	0.7 to 9 µg cm^−2^	1.4 to 6 µg cm^−2^	18(11.08)	29(6.81)	74(37.84)	84(22.53)	400(187.04)	159(84.36)	206(63)	150(187)	353(94)	146(101)	192(85.21)

SD is given in brackets. Concentrations that were previously reported [10], [12] for field collected apical tips (or whole algal individuals in the case of fucoxanthin) are also given, as well as previously reported necessary concentrations for halfmaximal inhibition (EC50) of various marine bacterial isolates [10], [12].

#### Quantitative analysis of biofilm

Epiphytic algae – mainly diatoms of the genus *Melosira* were observed in all treatments. Their density generally increased during the first three weeks of the experiment. Epiphytism increased with temperature: after three and four weeks the algae incubated at 5°C still appeared relatively clean (bearing only few macroscopically visible algal filaments), while those incubated at 25°C were massively overgrown. Temperature had no significant influence on bacterial cell density [6].

#### Qualitative analysis of biofilm

Altogether, pyrosequencing detected 4348 different OTUs that were associated with *F. vesiculosus* in the temperature experiment. More than 40% of them belonged to the Alpha-Proteobacteria, with Rhodobacteraceae alone contributing approximately 25% (Fig. S1). Other important taxa were Gammaproteobacteria, Flavobacteriaceae and Saprospiraceae (Fig. S1)

### Light experiment

#### General algal response

Symptoms of decay, as those observed in the temperature experiment, did not occur in the light experiment. Regression analysis showed that there was a significant positive relationship between available light energy and mannitol concentration in differently light treated individuals of *F. vesiculosus* (y = −0.025x+0.765, r^2^ = 0.88, P<0.05, Fig. S2).

#### Quantification of defence chemicals

The mean concentrations of DMSP and proline on surfaces of the apical branches of light treated algae were in the range of 0.03–0.45 ng cm^−2^ and 0.01–0.03 ng cm^−2^, respectively. Fucoxanthin concentrations on these branches ranged from 146 to 353 ng cm^−2^. As in the temperature experiment the variability of concentrations within treatments was considerable in some cases. The surface DMSP concentration varied among light intensities (1-way ANOVA, F = 4, p = 0.009, Tukey's HSD test, Fig. 1 (iv)), with particularly high concentrations under dark conditions (Table 1). In contrast, there was no significant variation of proline surface concentrations among differently light treated individuals (1-way ANOVA, F = 0.75, p = 0.59, Fig. 1 (v)). Fucoxanthin varied moderately among different light intensities, with the highest concentration at the 23% light regime (1-way ANOVA, F = 4, p = 0.01, Tukey's HSD test, Fig. 1 (vi)). See table 1 for details.

#### Quantitative analysis of biofilm

The diatom density did not differ significantly among the light levels (1-way ANOVA, F = 1.6, p = 0.17, Fig. S3). However, diatom density correlated positively with the concentration of fucoxanthin (y = 22.279+0.10376x, r^2^ = 0.92, p<0.05, Fig. S4). The total density of bacterial cells per cm^−2^ of algal surface did not differ significantly among the light levels (1- way ANOVA, F = 2.3, p = 0.06, Fig. S5).

#### Qualitative analysis of biofilm

Altogether, 3182 OTUs were associated with *F. vesiculosus* in the light experiment. Approximately 30%, 25% and 10% of them belonged to the family Flavobacteriaceae, Rhodobacteraceae and Saprospiraceae, respectively (Fig. S1).

#### Bacterial community composition vs. surface concentration of defence chemicals in both experiments

**Figure 2:**
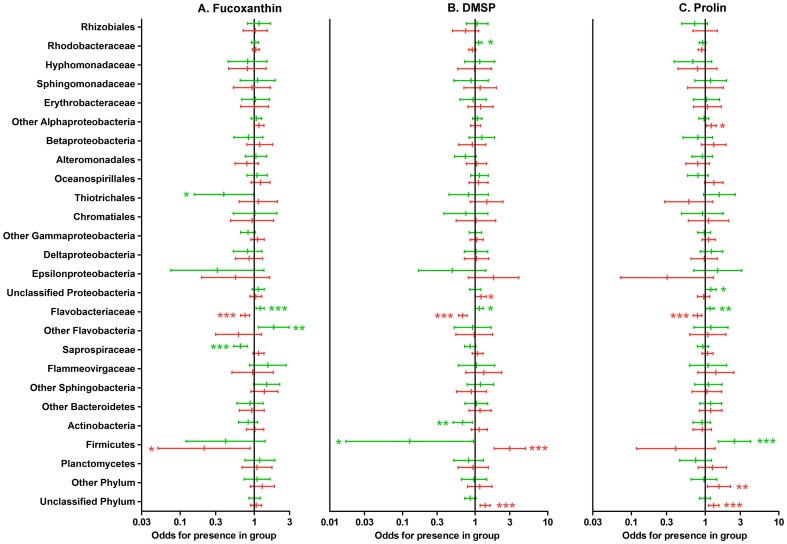
Odds for the presence of 26 different clades of bacteria within those groups of bacteria that consistently exhibited either a negative (red) or a positive (green) correlation between OTU abundance and concentration of Fucoxanthin (A), DMSP (B) and Proline (C). Geometric means of odds calculated for the temperature experiment and the light experiment ±95% confidence intervals. Asterisks indicate odds that are significantly different from 1 (χ^2^-test, * p<0.05, ** p<0.025, *** p<0.001).

The distribution of many of the higher phylogenetic taxa of bacteria was not significantly different among the total bacterial community and the six subgroups of deterrent-positive or deterrent negative OTUs (Fig. 2). However, some taxa were significantly over- or underrepresented in each of these subgroups; and this was in all cases not only indicated by the χ^2^ statistics but also by 95% confidence intervals that excluded 1 (Fig. 2). Among the bacteria that were Fucoxanthin-positive, Flavobacteriaceae and other Flavobacteria were overrepresented, as indicated by the significantly increased odds for their presence in this subgroup (Fig. 2a). At the same time, some Flavobacteria were significantly and other Flavobacteria were non-significantly underrepresented among the bacteria that were Fucoxanthin-negative. This suggests that Flavobacteria tend to be attracted by Fucoxanthin. In contrast, Saprospiraceae, Thiotrichales and Firmicutes were significantly underrepresented in the subgroup of Fucoxanthin-positive bacteria and therefore tend not to be attracted by fucoxanthin. However, none of these taxa exhibited significantly increased odds for presence in the subgroup of Fucoxanthin-positive bacteria. Thus, fucoxanthin affected the community composition through attraction of specific taxonomic groups, as an overall deterring effect towards taxonomic groups could not be detected.

In contrast, DMSP probably deterred Firmicutes, as they were clearly overrepresented in the subgroup of DMSP-negative and at the same time underrepresented in the subgroup of DMSP-positive bacteria (Fig. 2b). A similar pattern also emerged for unclassified phyla, although the odds for their presence in the DMSP-positive group were not significantly reduced (p<0.06). Also, unclassified Proteobacteria were overrepresented in the group of DMSP-negative bacteria. Evidence for a promoting effect of DMSP was apparent for the two most abundant families of bacteria that are associated with *F. vesiculosus*, the Flavobacteriaceae and the Rhodobacteraceae. Both were significantly overrepresented among DMSP-positive bacteria, and the Flavobacteriaceae were also underrepresented among DMSP-negative bacteria.

Flavobacteriaceae were overrepresented among Proline-positive and underrepresented among Proline-negative bacteria (Fig. 2c), suggesting that they also tend to be attracted by this compound. Also overrepresented in the Proline-positive subgroup were Firmicutes and unclassified Proteobacteria. In contrast, the odds for presence in the subgroup of Proline-negative bacteria were significantly increased for “other” ( = rare) Alphaproteobacteria, for “other” ( = rare) phyla and for unclassified phyla.

## Discussion

The surface concentrations of all three bacterial settlement inhibitors of *F. vesiculosus* varied considerably among temperature and light experiments. Despite this background variation DMSP and fucoxanthin concentrations also varied among different temperature and among different light conditions. In contrast, the concentration of proline was not significantly affected by different treatment levels. Different metabolite concentrations were also found under comparable factor settings in the two experiments, possibly resulting from the differences in experimental set-up and/or in season and/or in sampling site variation. Overall, DMSP and fucoxanthin were less concentrated in the light experiment, while the inverse was true for proline.

At least some of the treatment levels applied in both experiments clearly had direct effects upon *F. vesiculosus* that may have caused differences in algal defence chemistry. For example, in the temperature experiment severe stress - resulting in the decay of apical thallus parts - was obviously exerted when *F. vesiculosus* was incubated for more than one week at 25°C. Correspondingly, 20°C is considered as the highest surface water temperature that may pertain for several weeks in natural habitats of *F. vesiculosus* [3]. No such morphological symptoms of stress were observed in the light experiment. However, the mannitol concentration of *F. vesiculosus* decreased in a linear manner with decreasing light, indicating a significant limitation of photosynthetic CO_2_ fixation under low light conditions [3].

Certain marine heterotrophic bacteria are known to be attracted by DMSP [26] and some bacteria are known to metabolize DMSP quickly [27]. Likewise, various bacteria and other microorganisms have a capacity for uptake and metabolization of proline [28]. On the other hand, not only *F. vesiculosus*, but also diatoms and some other epiphytic algae are known to produce DMSP [29] and fucoxanthin [10]. Although the bacterial densities did not differ significantly among different temperature and among different light treatments, considerable variation in bacterial community composition was observed [6], [30] in the present study. For example, in the temperature experiment the relative abundance of the family Rhodobacteraceae varied between 20 to 50% among treatments and even more among single samples [6] and in the light experiment significant differences in terms of OTU richness were also detected [30]. In this light, variation in the concentrations of those compounds may not only result from light and temperature effects upon the host, but also from such effects upon associated microbial taxa that modulate their production or fate. Also the relatively high variability within treatment levels could result from differences in the bacterial community composition.

To date few studies have investigated the effect of temperature upon the production of defence chemicals by macroalgae. In previous studies with field collected material, DMSP has been detected on *Fucus* surfaces at concentrations in the range of 0.12 to 1.08 ng cm^−2^ [12]. In the present study, differently temperature treated samples of *Fucus* contained DMSP at similar concentrations, with the highest concentrations at 20°C (Fig. 1(i), Table 1). DMSP surface concentrations in *F. vesiculosus* were low at 10°C and at the highest temperature that was tested (25°C). The particularly low surface concentration of DMSP at 25°C despite particularly strong fouling by epiphytic diatoms indicates that DMSP was not contributed by these epiphytes. Overall, the surface DMSP concentration of *F. vesiculosus* was lower in the light experiment than in the temperature experiment (Table 1), with relatively high mean concentrations at 100% sun light and total darkness. A light limitation thus did not pose any hindrance to DMSP production in *F. vesiculosus*, although light reduction resulted in significantly reduced CO_2_ fixation. The variability at 100% was particularly high, while significantly less DMSP was detected on *F. vesiculosus* that was maintained at 44% of sun light, as compared to 13% and 0% (Fig. 1(iv)), which suggests that a certain tendency towards reduced presence of surface DMSP at high light may exist. Apparently in contrast, in *Codium fragile* [31] and many other algae [32], tissue DMSP usually increases with light intensity, often due to its ecophysiological role as an antioxidant. Also the increase of *Fucus* DMSP with temperature from 10°C up to 20°C contrasts to inverse observations with the green alga *Codium fragile* [31]. However, different from the mentioned reference studies we determined surface concentrations that are relevant for antifouling and not tissue concentrations of DMSP that are relevant for cryoprotection and management of oxidative stress. The relatively low concentration of DMSP under high temperature conditions corresponds with the environmental stress hypothesis (EST) and with a similar report for another defence compound, elatol (tissue concentration) in *Laurencia dendroidea* [33]. The EST predicts that the concentration of defensive compounds can decrease in stressed organisms [34]. On the other hand, the tendency for high concentration of DMSP under light limitation seems not to support the EST.

DMSP has previously been shown to inhibit the settlement of four out of five potential bacterial microfouler strains (*Bacillus aquimaris*, *Ulvibacter littoralis*, Alteromonadaceae E1 and marine sediment bacterium ISA 7311; all isolated from rockweed dominated habitats in the Baltic Sea) at a surface concentration of 0.05 ng cm^−2^ or more. The fifth strain *Cytophaga sp.*; also isolated from rockweed dominated habitats, required a higher DMSP concentration of 0.38 ng cm^−2^ for 50% inhibition [12]. Based upon the mean concentrations that were detected in our study, the former four strains would be largely inhibited by DMSP at all tested temperature conditions - including 25°C – and at all tested light regimes (Table 1). In contrast, *Cytophaga sp.* would be largely repelled by DMSP at a lower temperature of 5°C and at intermediate temperatures of 15 and 20°C, as well as in complete absence of light – a response that could also be expected from other microfouler bacterial strains.

In a former study proline was detected on *Fucus* apical tips at concentrations in the range between 0.09 and 0.59 ng cm^−2^ [12]. In the present temperature and light treatment experiments proline was detected at much lower concentrations (Table 1) with no significant differences among the treatments (Fig. 1(ii) and 1(v), Table 1). Proline concentrations of 0.01 ng cm^−2^ or more, sufficient for a partial inhibition of all but the most resistant strain *Cytophaga* sp. [12], were present on the surface of *F. vesiculosus* under all conditions in the light experiment, but only at 15°C in the temperature experiment.

Fucoxanthin has been detected previously at a concentration of 0.7 µg cm^−2^ on *Fucus* apical tips [10]. But considerably lower surface concentrations were detected on the younger algal branches that had been subjected to the temperature treatments described here. Epiphytic diatoms which contribute to the fucoxanthin accumulation at the surface of *F. vesiculosus* [10] were particularly abundant at 25°C and relatively rare at 5°C. Likewise, in the light treated algae a significantly higher concentration of fucoxanthin as well as a particularly high abundance of diatoms was detected under 23% of sunlight. Diatoms apparently contributed an important amount of surface fucoxanthin, as fucoxanthin concentration correlated significantly and positively to diatom abundance in both experiments.

Fucoxanthin surface concentrations of 1.4 to 6 µg cm^−2^ detected in a former investigation caused a 50% settlement inhibition in four out of five bacterial test strains (*Ulvibacter littoralis*, AlteromonadaceaeE1, *Cytophaga* sp. and *B. aquimaris*), while the fifth isolate (marine sediment bacterium ISA 7311) showed a similar effect only above 6 µg cm^−2^ [10]. In the experiments conducted here fucoxanthin was always detected at concentrations below 6 µg cm^−2^. Only a feeble – if any - contribution of fucoxanthin to antisettlement defense can, thus, be assumed based upon its concentrations in both experiments.

Overall DMSP appeared as the most relevant defence metabolite in both the temperature and light treatment, as it was detected at sufficient concentrations for moderate to strong inhibition of relevant bacterial microfoulers at all tested temperature and light levels. Proline seemingly contributed to this defence in the light experiment only, fucoxanthin under none of the test conditions. Interestingly, this picture – deduced from the sensitivities of single bacterial isolates - was to some degree reflected in the relative abundance of bacterial taxa within total bacterial communities: OTU's that tended to be more abundant on specimens exhibiting lower surface concentrations of DMSP were overrepresented within three different bacterial groups and the same was detected for two groups with respect to proline, but in no case for fucoxanthin (Fig. 2). Thus, in the current study fucoxanthin played no apparent role for the deterrence of microfoulers.

Bacteria that appeared as attracted rather than deterred by DMSP were overrepresented among the Rhodobacteraceae and Flavobacteriaceae, two Families which alone contribute 25% and 8 to 30% of all OTU's of the *F. vesiculosus* microbiome, respectively. Attraction of heterotrophic bacteria by DMSP has been previously reported [27] and in addition to deterrence this attraction apparently also plays a significant role in the shaping of *Fucus*-associated bacterial communities. Attraction by fucoxanthin and proline was reflected in terms of overrepresentation among Flavobacteriaceae. Similar as Rhodobacteraceae, Flavobacteriaceae are generally abundant on seaweeds [35] and they seemingly include many taxa that are well adapted to the defence compounds that are present on those hosts. In contrast, Firmicutes – that are relatively rare on *Fucus* – at the same time appear as sensitive towards DMSP. Interestingly the groups of unidentified Alphaproteobacteria and unidentified Phyla also harboured numerous taxa that appear as sensitive towards DMSP and proline.

In conclusion, the defence system of *F. vesiculosus* against bacterial foulers is composed of at least three metabolites which exhibit variable concentrations. However, the effect of light and temperature – upon these concentrations appears as limited - even under conditions clearly stressful for the algal host. Moreover, under all tested conditions the natural surface concentration of at least one of the defensive metabolites was high enough to warrant for a reduction of bacterial settlement. Thus, while the concentration of single compounds may be occasionally reduced under certain adverse conditions –the overall antifouling defence of *F. vesiculosus* appears not to be significantly affected by light limitation stress or disruptive temperature stress, including complete absence of light for 18 d and 25°C for 4 weeks. Complex defence systems composed of several (independent) mechanisms may not only make the antifouling defence more efficient and less vulnerable to co-adaptation as suggested earlier [36] but also less sensitive to environmental stress. In addition, additive or synergistic action of multiple defence compounds could make them more efficient. For example, the polar compounds proline and DMSP probably act additively or synergistically. When total polar surface extract of *F. vesiculosus* was tested without prior fractionation and purification it had a stronger inhibitory effect on bacterial settlement than DMSP and proline alone, which could be explained through an additive effect among the identified or even among unidentified components (see [10] for details).

## Supporting Information

Figure S1 **Relative contribution [%] of major phylogenetic groups to the overall composition of bacterial communities associated with** ***F. vesiculosus*** **in the temperature and the light treatment.**
(TIF)

Figure S2 **Relationship between tissue mannitol concentration and light among differently light treated individuals of** ***F. vesiculosus*** **(r^2^ = 0.880, p<0.05).** Straight line: best fitting linear function (y = −0.025x+0.765).
(TIF)

Figure S3 **Variation of diatom abundance among differently light treated** ***F. vesiculosus*** **individuals.** Median (central symbol), n = 10, interquartile range.
(TIF)

Figure S4 **Relationship between surface fucoxanthin concentration and relative diatom abundance cm^−2^ of algal surface among differently light treated individuals of** ***F. vesiculosus*** **(r^2^ = 0.92, p<0.05).** Straight line: best fitting linear function (y = 22.279+0.10376*x). Dotted lines: 95% CI.
(TIF)

Figure S5 **Variation of bacteria abundance among differently light treated** ***F. vesiculosus*** **individuals.** Median (central symbol), n = 9, interquartile range.
(TIF)
