# Supplementary figures and images for: Defence Chemistry Modulation by Light and Temperature Shifts and the Resulting Effects on Associated Epibacteria of Fucus vesiculosus
Source: PLoS One. 2014 Oct 31;9(10):e105333. doi: 10.1371/journal.pone.0105333 (PMC4215838; doi:10.1371/journal.pone.0105333)

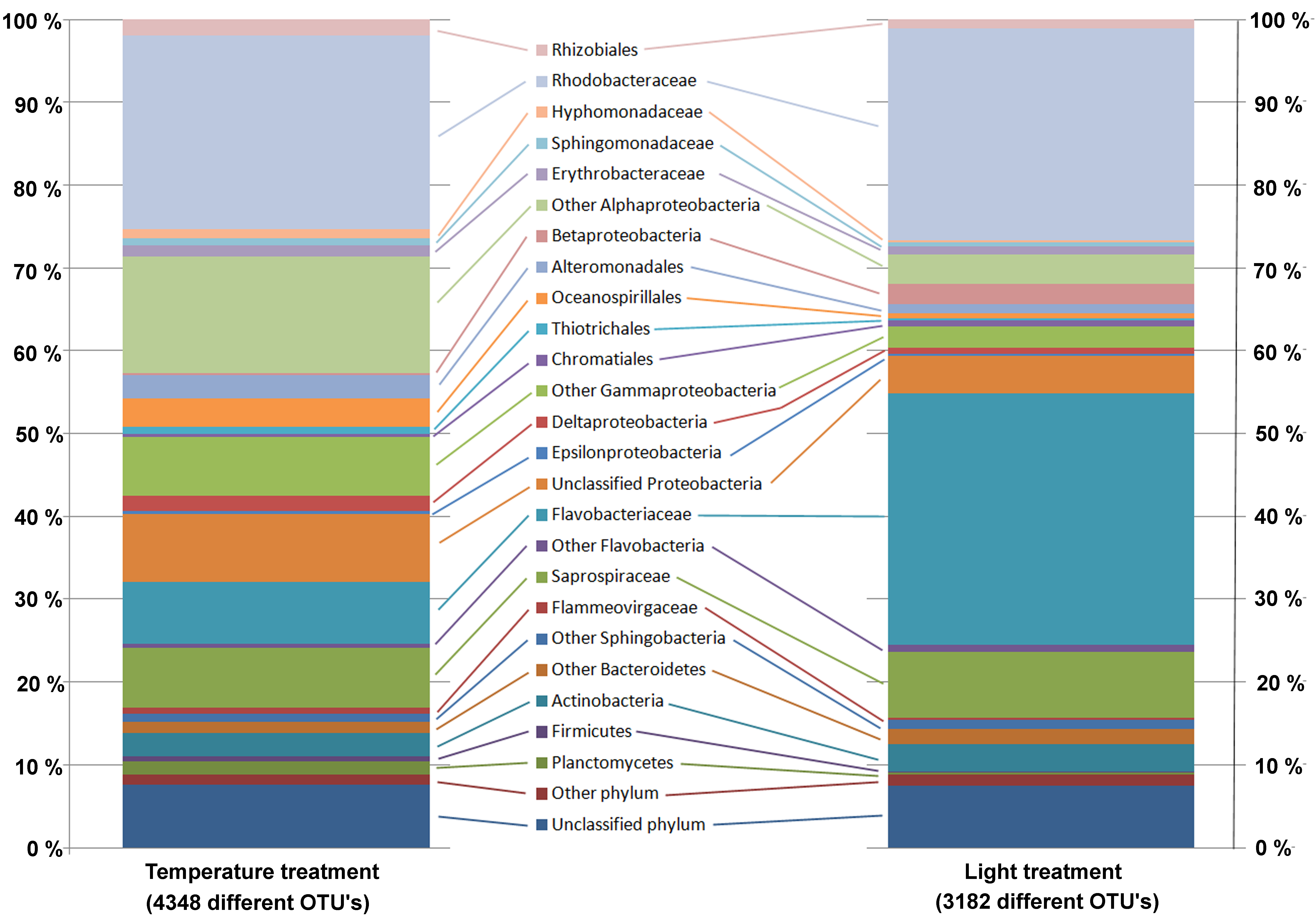

Supplement: Figure S1 — Relative contribution [%] of major phylogenetic groups to the overall composition of bacterial communities associated with F. vesiculosus in the temperature and the light treatment. (TIF) [file pone.0105333.s001.tif]

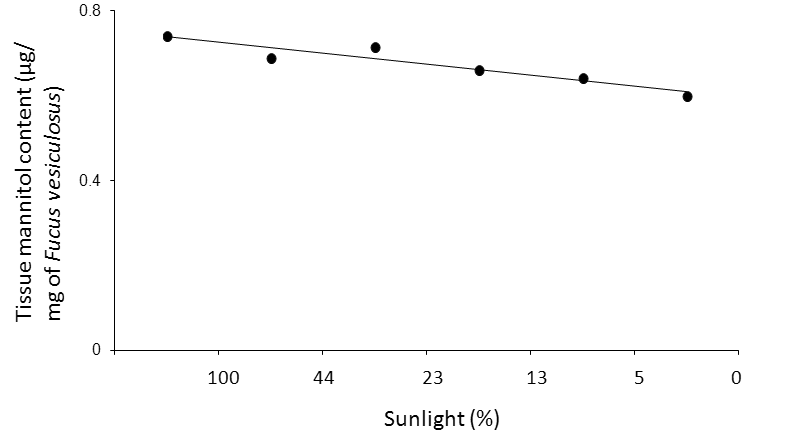

Supplement: Figure S2 — Relationship between tissue mannitol concentration and light among differently light treated individuals of F. vesiculosus (r2 = 0.880, p<0.05). Straight line: best fitting linear function (y = −0.025x+0.765). (TIF) [file pone.0105333.s002.tif]

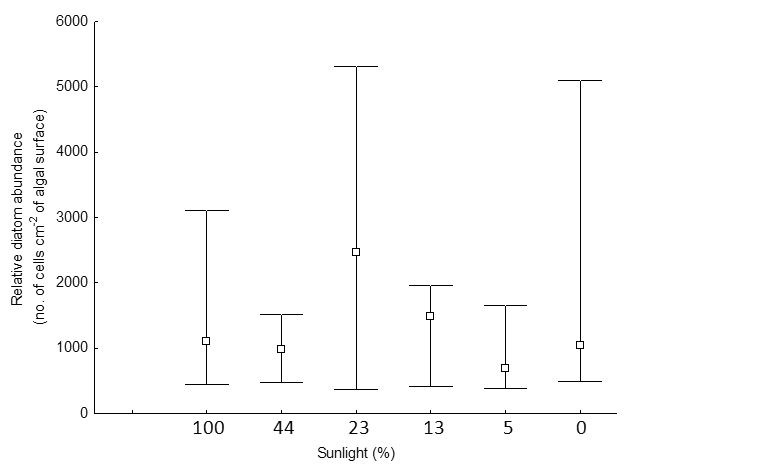

Supplement: Figure S3 — Variation of diatom abundance among differently light treated F. vesiculosus individuals. Median (central symbol), n = 10, interquartile range. (TIF) [file pone.0105333.s003.tif]

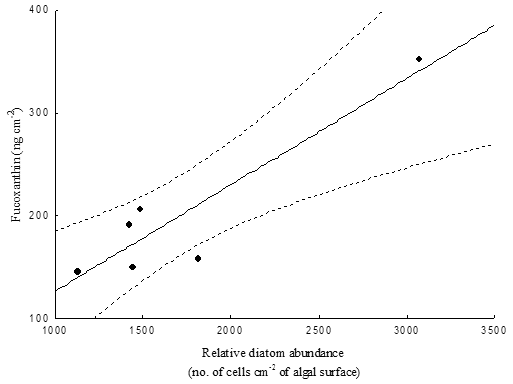

Supplement: Figure S4 — Relationship between surface fucoxanthin concentration and relative diatom abundance cm−2 of algal surface among differently light treated individuals of F. vesiculosus (r2 = 0.92, p<0.05). Straight line: best fitting linear function (y = 22.279+0.10376*x). Dotted lines: 95% CI. (TIF) [file pone.0105333.s004.tif]

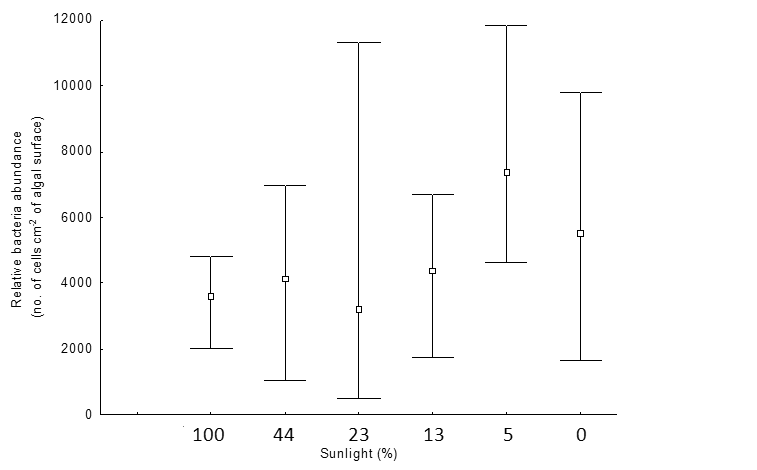

Supplement: Figure S5 — Variation of bacteria abundance among differently light treated F. vesiculosus individuals. Median (central symbol), n = 9, interquartile range. (TIF) [file pone.0105333.s005.tif]
